# A Trusted Reputation Management Scheme for Cross-Chain Transactions

**DOI:** 10.3390/s23136033

**Published:** 2023-06-29

**Authors:** Kuongho Chen, Lin-Fa Lee, Wayne Chiu, Chunhua Su, Kuo-Hui Yeh, Han-Chieh Chao

**Affiliations:** 1 ezPay Co., Ltd., Taipei 11578, Taiwan; tek.chen@ezpay.com.tw; 2 Department of Information Management, National Dong Hwa University, Hualien 97401, Taiwan; 611035111@gms.ndhu.edu.tw; 3 Department of Applied Mathematics and Computer Science, Technical University of Denmark, 2800 Lyngby, Denmark; weich@dtu.dk; 4 Division of Computer Science, University of Aizu, Fukushima 965-0006, Japan; chsu@u-aizu.ac.jp; 5 Department of Computer Science and Engineering, National Sun Yat-sen University, Kaohsiung 80424, Taiwan; 6 Institute of Computer Science and Innovation, UCSI University, Kuala Lumpur 5600, Malaysia

**Keywords:** blockchain, cross-chain, reputation management system, interoperability, relay chain

## Abstract

Blockchain has become a well-known, secured, decentralized datastore in many domains, including medical, industrial, and especially the financial field. However, to meet the requirements of different fields, platforms that are built on blockchain technology must provide functions and characteristics with a wide variety of options. Although they may share similar technology at the fundamental level, the differences among them make data or transaction exchange challenging. Cross-chain transactions have become a commonly utilized function, while at the same time, some have pointed out its security loopholes. It is evident that a secure transaction scheme is desperately needed. However, what about those nodes that do not behave? It is clear that not only a secure transaction scheme is necessary, but also a system that can gradually eliminate malicious players is of dire need. At the same time, integrating different blockchain systems can be difficult due to their independent architectures, and cross-chain transactions can be at risk if malicious attackers try to control the nodes in the cross-chain system. In this paper, we propose a dynamic reputation management scheme based on the past transaction behaviors of nodes. These behaviors serve as the basis for evaluating a node’s reputation to support the decision on malicious behavior and enable the system to intercept it in a timely manner. Furthermore, to establish a reputation index with high precision and flexibility, we integrate Particle Swarm Optimization (PSO) into our proposed scheme. This allows our system to meet the needs of a wide variety of blockchain platforms. Overall, the article highlights the importance of securing cross-chain transactions and proposes a method to prevent misbehavior by evaluating and managing node reputation.

## 1. Introduction

After Nakamoto Satoshi delivered the first well-known cryptocurrency in 2008 [1], the underlying data storage that supports the currency’s state security and integrity in a decentralized and zero-trust manner has drawn much attention, that is, blockchain. It demonstrates a feasible way of reaching secure and cohesion data storing and distribution without any trust parties. Platforms built on this technology benefit from four major strength that blockchain provides [2], leading to the widespread adoption of blockchain technology in various fields:

Decentralization: In a blockchain, there is no central authority controlling everything. Instead, all the participants work together to maintain and update the ledger. Every transaction is shared with the entire network, eliminating the need for a central entity.

Anonymity: Blockchain transactions are represented by random-looking wallet addresses, not by real identities. Without a specific system in place to connect addresses to individuals, it is difficult to know who is behind a transaction unless they reveal their identity through other means.

Non-repudiation: Each block in the blockchain contains information about the previous block, thus forming a chain. Additionally, each block is securely sealed with a proof that shows it has been agreed upon by the network. This makes it highly impractical and time-consuming to change or tamper with existing transactions. Altering one transaction would require redoing all the subsequent blocks.

Consensus: Similar to non-repudiation, consensus mechanisms ensure that all participants in the blockchain network agree on the validity and order of transactions. This agreement is reached through various methods. The consensus process makes it difficult for someone to alter transactions because they would need to redo subsequent blocks and convince the network to accept their changes.

The development of decentralized applications (DAPPs) has also been made possible by the advent of smart contracts. However, as the number of DAPPs increases, two critical issues have emerged [3,4,5,6]. Firstly, blockchain systems are not immune to vulnerabilities when operating in real-life scenarios. Secondly, the unique architecture of each blockchain system creates difficulties in cross-blockchain communication, posing a challenge for interoperability between different blockchains in various industries, such as healthcare and finance. For example, hospitals using blockchain technology to maintain health records cannot modify information from other hospitals that also use blockchain as their database.

Each block in a blockchain contains the hash value of the previous block, each secured using a consensus proof based on the underlying algorithm [3,5]. This makes it highly difficult and time-consuming to alter existing transactions. If someone wants to change a transaction, they would need to redo not only the current block but also all the blocks connected after it. However, when it comes to interacting between different blockchains, there are challenges. Jin et al. [7] have identified security issues in cross-chain operations, such as ensuring reliable data access between the source and destination chains. Data integrity must be maintained during the off-chain transfer before it reaches the destination chain. Moreover, the destination chain needs to validate and agree on the transaction in a way that both the source and destination can trust, reaching a consensus on data access and storage. However, this is not easy to achieve due to the different architectures and consensus algorithms used in various blockchain platforms. These differences create security vulnerabilities during cross-chain operations, including denial-of-service attacks [8], double-spending issues [9], and selfish mining attacks [10].

Motivation: It is crucial to find a secure solution to support and enhance the security of cross-chain interoperability processes. The presence of various blockchain platforms emphasizes the need for proposed methods to be adaptable to the diverse blockchain landscape. Since transactions occur between chains through nodes, the trustworthiness of these nodes significantly affects the integrity and security of the transactions. Hence, there is a pressing need for a decentralized and self-evaluating mechanism in cross-chain systems, which can effectively reduce misconduct by potential nodes. Similar approaches have been seen in Rodrigues et al. [11], who used smart contracts for cooperative signaling to mitigate malicious network behavior, and Chai et al. [12], who employed proof-of-reputation to enhance the security of the Internet of Vehicles. While reputation management in information security has seen progress as more systems are digitized, there is a lack of research in emerging areas where reputation management is needed. For example, the increasing popularity of decentralized exchanges and inter-chain operability due to various cryptocurrencies has not been extensively explored in the context of cross-chain reputation management. To the best of our knowledge, He et al. [13], Xiong et al. [14], and Lee and Yeh [15] are the three papers that focused on the cross-chain reputation issue. Others have proposed methods dedicated to achieving cross-chain operability.

Compared to our previous work [15], in response to the need for a reputation management system that optimizes on the fly, we aim to prevent malicious nodes and behaviors as effectively as possible. Our focus is on accurately evaluating the trustworthiness of nodes and chains. We have chosen Particle Swarm Optimization (PSO) [16] as the base algorithm for reputation evaluation optimization. PSO offers several advantages:

- Simplicity: PSO is easy to understand and implement.
- Agility: PSO is known for its effectiveness in optimization with low computational cost.
- Continuity: PSO can handle optimization in discontinuous function spaces, which are often encountered in the design of separation and other networks.

Contribution: This paper presents a cross-chain reputation management scheme that aims to identify and exclude malicious attackers while evaluating the trustworthiness of nodes and chains. To address multiple threats, we utilize the Particle Swarm Optimization (PSO) heuristic algorithm [17] due to its advantages of low computing cost and optimizing discontinuity. We adopt seven indicators, identified through a survey of known attack methods, to ensure the security and accuracy of cross-chain interoperability. Each indicator corresponds to negative impacts on nodes and chains during or after a transaction and serves as a significant evaluation criterion for rating the reputation of nodes involved in cross-chain transactions. The main contributions of this paper are as follows:

- We propose a cross-chain reputation management system operating on a relay chain that manages entity scores and reputation weights automatically and appropriately. Furthermore, we discuss the effectiveness and weighting of indicators against specific malicious attacks on blockchains.
- The integration of the PSO algorithm allows for on-the-fly adjustments and provides flexibility and quick response without extensive computational resources. By dynamically reweighting reputation weights using the low computational consumption of the PSO algorithm, the scheme can adapt to different attack frequencies in cross-chain or single-chain transactions.
- The scheme’s process for judging honest nodes considers the transaction’s past reputation over a period of time. We integrate multiple weighting indicators, chosen based on recognized attack techniques that pose threats during or after transactions. Consequently, the scheme ensures a high degree of honesty among nodes in the cross-chain system by minimizing the presence of misbehaving nodes during transactions.

The rest of this paper is organized as follows: Related works in Section 2, proposed scheme in Section 3, experimental setting in Section 4, experimental results in Section 5, and conclusions in Section 6.

## 2. Related Work

In this section, surveys concerning PSO algorithm, blockchain reputation management system, and vulnerabilities of blockchain interoperation are presented.

### 2.1. Particle Swarm Optimization

Particle Swarm Optimization is a heuristic algorithm for solving the optimization problem which is based on the observations of biological social behavior. This algorithm was first proposed as an analogy to the flight patterns of migratory birds, and it was inspired by self-recognition and group behavior. The individuals in the algorithm are called particles and each particle is a solution agent. In PSO, each particle will have a certain velocity which will be dynamically modified according to its own or the swarm’s performance. In summary, particles will compute the best solution using Equations (1) and (2), which means updating velocity and particle vector, where, $v_{id}$ represents the velocity of particle i in optimization problem with d dimension, $x_{id}$ represents the position of particle i in optimization problem with d dimension, w is the inertia weight in optimization problem, $C_{1}$ and $C_{2}$ are acceleration constants used to refine the performance about PSO, $P_{id}$ means the best fitness value of particle i, $P_{gd}$ means the best fitness value of global swarm, and r is random number between 0 and 1.

(1) $$v_{id_{new}}=w\times v_{id}+C_{1}\times r\times \left(P_{id}-x_{id}\right)+C_{2}\times r\times \left(P_{gd}-x_{gd}\right)$$

(2) $$x_{id\_new}=x_{id}+v_{id\_new}$$

### 2.2. Blockchain Reputation Management System

Many reputation management systems (RMS) have been proposed to reduce or prevent attacks in blockchain. For instance, ref. [18] proposed a blockchain signaling system (BloSS) based on a reputation scheme that uses a smart contract-enabled process to automate reputation management, and it can diminish malicious behavior. BloSS presents a relatively basic and easy-to-implement RMS that uses mutual evaluation of actions to determine the credit earned after a transaction is completed. The earned credit follows a node for its lifetime, enabling other nodes to decide whether to interact with it based on its reputation score. However, BloSS only provides protection against DDoS attack and may not be sufficient for real-world implementation. In another study [12], a novel blockchain framework for resource sharing and trust establishment in the Internet of Vehicles (IoV) using a proof-of-reputation (PoR) consensus mechanism was proposed to improve the capability of security in the IoV when sharing among vehicles. The objective of PoR is to minimize the computational power required and incentivize the vehicles involved in resource sharing. This paper demonstrates that the reputation management system can be implemented in real-life scenarios. However, there is a huge cost associated with designing a specific method for each scenario. Therefore, it is necessary to design a universally applicable RMS to protect multiple blockchains and defend against multiple attack techniques. Moreover, the use of RMS in cross-chain interoperability has received relatively few academic contributions.

On the other hand, Dennis et al. [19] proposed a reputation system based on previous interactions, while RBFT [20] uses an extra data structure to determine the reputation of nodes in the Byzantine fault tolerance (BFT) consensus process. BARS [21] utilizes blockchain technology and zero-knowledge proofs for anonymous reputation management, and has demonstrated improved accuracy and resilience against attacks compared to existing approaches. In [22], a reputation method was proposed for Internet of Vehicle (IoV) scene, where the reputation of road side units (RSUs) is evaluated based on data accuracy and stability of the connection during vehicle-to-vehicle data sharing process. In [23], a decentralized IoT public fog nodes reputation system based on Ethereum blockchain and smart contracts was proposed, which can manage reputation scores for a large number of IoT devices in a transparent and tamper-proof manner. These systems have shown promising results in various scenarios, and offer potential solutions for reputation management in decentralized and secure manner.

### 2.3. Blockchain Transaction Vulnerabilities

Several types of attacks/vulnerabilities are detailed as follows:

- Stale and orphaned blocks: When two miners finish mining at the same time, two blocks are created simultaneously, causing a fork in the blockchain, and only one block will be selected to continue working with, and the other would be discarded, thus causing blockchain instability [24].
- Selfish mining: As explained in [25], certain miners opt for this strategy to increase their rewards by keeping their block private, resulting in honest miners losing their rewards, which then results in consensus delays, blockchain forks, or double-spending attacks.
- Block withholding attacks: Miners in a pool who find a block can keep the hash value and not broadcast it, causing a loss to the mining pool. It can be detected by the delay time of consensus process or transaction [26].
- Consensus delay: A type of attack associated with the blockchain’s network architecture, in which an attacker sends false blocks to increase or prevent peers from reaching consensus. It can be detected by examining the result of the last transaction verification and the time spent on sending blocks [3].
- Time-jacking attack: Imprecise timestamps within the blockchain can lead to a time-jacking attack in which an adversary continuously sends blocks with false timestamps, causing the target node to reject new blocks, leading to its removal from the blockchain network [27].
- Double spending: The infinite replication of digital assets in the blockchain network can cause an asset to be spent repeatedly. Before a transaction confirms, it can be backtracked and revalidated or even replaced by another transaction due to temporary or malicious forks in the blockchain [28].
- DDoS attack: Attackers send a large number of useless transactions to the blockchain, disrupting network resources and reducing the success rate of transactions [29].

## 3. Proposed Scheme

In this section, we introduce our proposed dynamic reputation management scheme which is extended from [23]. The relay chain is responsible for the agreement of transaction and the corresponding consensus which will be uploaded and accepted in the blockchain systems.

Basically, we have a major relay chain and two sub-chains in our system scenario. Each blockchain system and each node will have a specific reputation value as the degree of trust in the next interactions (and transactions). As the proposed system is used to prevent the misbehavior in heterogeneous blockchains, the analysis of the node and the chain’s past normal transaction records is adopted to detect potential misbehaviors. In addition, the proposed system allows to dynamically modify indicator weights by nodes in the relay chain according to the frequency of current misbehaviors.

Reputation indicators in Table 1 are adopted in the proposed method.

As a candidate for reputation evaluation, algorithm has been selected out, and for the next step we look for the indicators for evaluation. After surveying the attacks on transactions among a variety of blockchain platforms, we select the indicators presented in Table 1. The implementation of reputation indicators is essential for detection of various malicious behavior prevalent in the common blockchain attack method.

- (Node-level indicator) Hardware utilization (GPU): Abrupt changes in a node’s GPU usage or hash power may indicate potential threats, such as selfish mining, block withholding, or majority attacks. We expect nodes within the system to maintain stable mining power to ensure smooth operation. As these threats often involve anomalous use of computational resources, it can compromise the network’s stability. Therefore, we monitor the average GPU computational power of nodes over a certain period and assign negative scores when drastic changes occur.
- (Node-level indicator) Average spending time of transaction: Unstable average transaction times could provide attackers with opportunities to initiate double-spending attacks or make consensus delay. These attacks often involve multiple verifications and confirmations of a transaction, potentially leading to transaction uncertainty or blockchain security issues. Consequently, if the transaction completion time does not meet expectations, the system will assign negative scores to the nodes involved.
- (Node-level indicator) Transaction consequence: Attacks, such as DDoS, double-spending, or time-jacking, can directly impact the transaction result. While not all transaction failures can be attributed to these attacks, in a stable cross-chain system, it is expected that nodes will complete transactions successfully after initiation. Therefore, the system will assign negative scores to nodes when transaction failures occur and positive scores when transactions are completed successfully.
- (Chain-level indicator) Average network hash rate: Similar to the node’s GPU usage, drastic changes in the hash rate could indicate the likelihood of various threats, such as stale and orphan blocks, selfish mining, or majority attacks. These threats can significantly impact the cross-chain system’s overall performance and stability, resulting in a negative evaluation for the involved single blockchain network.
- (Chain-level indicator) The delay time in block propagation: Delays in block propagation can affect the speed and security of consensus, providing opportunities for various potential attacks. Delays can reveal issues, such as consensus delays, selfish mining, block withholding, stale and orphan blocks, or time-jacking attacks. Thus, when a network experiences delays in block propagation, the single blockchain responsible for the delay is assigned a negative score.
- (Chain-level indicator) Average spending time of each transaction: Similar to the average transaction time of nodes, the system also monitors this metric for each single blockchain in the system to anticipate potential uncertainties or security issues in the transactions. Consequently, if the transaction completion time deviates from expectations, the single blockchain is assigned a negative score.

The pre-defined weights for each indicator are determined based on the number of corresponding attacks and threats. Please note that these weights may vary in different scenarios. After our proposed indicator reweight method, each indicator will obtain a weight that is most suitable for the current cross-chain scenario.

### 3.1. Cross-Chain Interoperation Process

**Figure 1:**
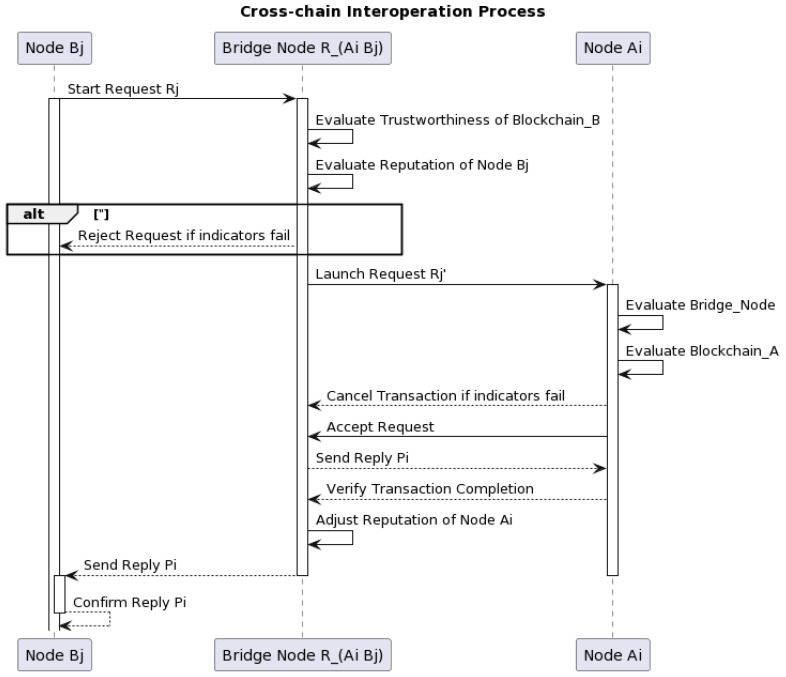
The cross-chain interoperation process.

As shown in Figure 1, suppose that $node\;B_{j}$ in *blockchain_B* wants to deploy a smart contract to exchange information with $node\;A_{i}$ in *blockchain_A*.

The details of the phases are presented as follows.

Phase 1 Node $B_{j}$ starts a request $R_{j}$ to a bridge node $R_{A_{i}B_{j}}$, which is responsible for transaction exchanging between nodes $A_{i}$ and $B_{j}$, in the relay chain. The bridge node $R_{A_{i}B_{j}}$ will establish a secure channel to *blockchain_B* and launch a cross-chain interoperation.
Phase 2 Node $R_{A_{i}B_{j}}$ will judge the trustworthiness of *blockchain_B* in terms of the reputation value through the three chain-level indicators, i.e., average network hash rate, the delay time in block propagation and average spending time of each transaction, as shown in Table 1. Meanwhile, node $R_{A_{i}B_{j}}$ will evaluate if the reputation of node $B_{j}$ is satisfied through the four node-level indicators, i.e., node connect status, hardware usage, average spending time of transaction and transaction consequence, presented in Table 1. If one of these seven indicators does not pass a pre-defined threshold, node $R_{A_{i}B_{j}}$ will be judged as a potentially misbehaved node. The incoming request $R_{j}$ will be rejected and node $R_{A_{i}B_{j}}$ will send a message to node $B_{j}$ as a termination command. If all of these seven indicators are passed, it will proceed to Phase 3.
Phase 3 Node $R_{A_{i}B_{j}}$ then launches a request $R_{j}\prime$ and sends $R_{j}\prime$ to node $A_{i}$. At the same time, the trustworthiness of node $A_{i}$ and *blockchain_A* will be evaluated through the same steps in Phase 2. That is, the seven indicators presented in Table 1 will be adopted to examine whether node $A_{i}$ and *blockchain_A* is classified as misbehaved one or not.
Phase 4 Similarly, based on the indicators, node $A_{i}$ then evaluates if the reputation of node $R_{A_{i}B_{j}}$ is satisfied after obtaining the request $R_{j}\prime$. If it is not satisfied, node $A_{i}$ will send a message to node $R_{A_{i}B_{j}}$ to cancel the current transaction. Otherwise, node $A_{i}$ will accept the request. Next, node $A_{i}$ will send a reply $P_{i}$ to node $R_{A_{i}B_{j}}$.
Phase 5 Node $R_{A_{i}B_{j}}$ will then check whether node $A_{i}$ has successfully completed the request after receiving reply $P_{i}$. In case of a normal transaction (which is successfully completed), the reputation of node $A_{i}$ will be adjusted and reply $P_{i}$ will be sent back to node $B_{j}$ through node $R_{A_{i}B_{j}}$. Otherwise, the reputation of node $A_{i}$ will be adjusted and the transaction will be terminated.
Phase 6 Node $B_{j}$ confirms reply $P_{i}$ and the cross-chain transaction will be considered as a finished one. In case of a failed transaction, the reputation of node $R_{A_{i}B_{j}}$ will be adjusted by node $B_{j}$. Afterwards, the information related to the failed transaction will be reported by node $B_{j}$.

### 3.2. Indicator Reweight Process

**Figure 2:**
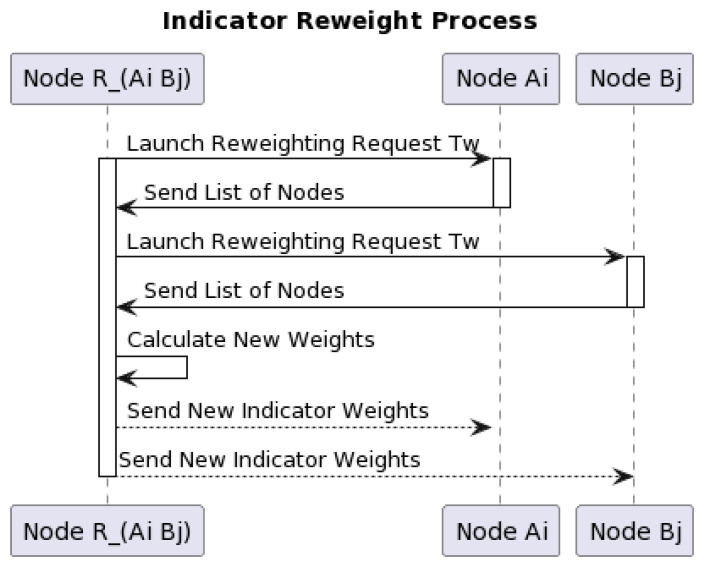
The example of cross-chain indicator reweight process.

Through the combination of PSO, the indicator weights in cross-chain system enable to cope with different frequency of misbehavior. We assume that a single node in the blockchain is a particle in the PSO algorithm. The new indicator weight is taken from the best solution of the single node or the global best solution of the blockchain. In other words, each node in node list will propose the best indicator weight they consider. As shown in Figure 2, the indicator reweight process occurs when relay chain considers that the frequency of misbehavior in cross-chain system has exceeded the default threshold.

At this point, the relay chain launches a cross-chain transaction for the purpose of re-weighting each indicator’s weight. The details of the phases are presented as follows:

Phase 1 Node $R_{A_{i}B_{j}}$ launch cross-chain re-weighting request $T_{w}$ to node $A_{i}$ in *blockchain_A* and node $B_{j}$ in *blockchain_B* with secure channel at the same time. Suppose node $R_{A_{i}B_{j}}$ is responsible for this re-weighting process, which also selects node $A_{i}$ and node $B_{j}$ based on the past reputation.
Phase 2 Node $A_{i}$ and node $B_{j}$ will determine and send a list of nodes to node $R_{A_{i}B_{j}}$ after relay chain receive the request $T_{w},$ and then the assignment node $R_{A_{i}B_{j}}$ lead to this indicator reweight process.
Phase 3 $Node\;R_{A_{i}B_{j}}$ receives the list of nodes, and the calculation of the new weight will be started. This weight is computed according to fitness function (3) and Equations (4) and (5) of the standard PSO algorithm. We believe that reputation management systems should be designed to maximize the benefits of the nodes in the system than the risks. Hence, computing node which participates in the indicator reweight process will compute the best solution by the fitness function as show in (3). MS represents the number of successful computing node transactions completed in the past. MF represents the number of failed transactions in the computing node’s blockchain due to misbehavior in the past. After that, computing node will find the next round j-th indicator weight in our indicator list using Equations (4) and (5). The process will continue until all indicators have been computed; w is the weight given to the degree of the previous modify, $v_{w}[j]$ represents the range of modify of the j-th indicator in the previous round, $P_{id}[j]$ means the best weight computed from the computing node for the j-th indicator, $x_{id}[j]$ means the past best weight computed from the computing node for the j-th indicator, $P_{chain}[j]$ represents the new global best weight of the blockchain in which the computing node is located, and $x_{chain}[j]$ means the past global best weight of j-th indicator from the blockchain in which the computing node is located.
  (3) $$Fitness=Fitness+\left(\frac{MS}{MS+MF}-\frac{MF}{MS+MF}\right)$$
  (4) $$v_{w\prime }[j]=w\times v_{w}[j]+C_{1}\times r\times \left(P_{id}[j]-x_{id}[j]\right)+C_{2}\times r\times \left(P_{chain}[j]-x_{chain}[j]\right).$$
  (5) $$x_{new\_weight}[j]=x_{chain}[j]+v_{w\prime }[j].$$
Phase 4 Node $R_{A_{i}B_{j}}$ will consider the outcome as a finished one and send the result to node $A_{i}$ and node $B_{j}$ on two sub-chains. Then, node $A_{i}$, node $B_{j}$ and node $R_{A_{i}B_{j}}$ will broadcast the new indicator weight in which the blockchain is located. After that, the reputation of the nodes involved in this indicator reweight process will be increased.

## 4. Experimental Implementation

Table 2 presents the detailed implementation environment of our prototype cross-blockchain system, which comprises both hardware resources and software platforms. To program our smart contract, we employed Remix and Solidity. Upon compilation via Remix, we utilized Web3j CLI to convert the application binary interface (ABI) and source code (bytecode) files to Java code. In order to monitor the state of each blockchain, we developed a decentralized application (DAPP) in Java within the Eclipse 3.8 environment, which was then integrated into Web3j CLI. The DAPP monitor provides information on the reputation of chains, transaction nodes, and mining nodes, as well as relevant indicators for each transaction. Furthermore, the DAPP serves as a trigger for the indicator reweight process, alerting the relay chain of any misbehavior or failed transactions, thus enabling prompt indicator reweighting. Additionally, the DAPP offers a PSO API to relay nodes, enabling them to make calls and assist in metrics reweighting. All of the experiment setting and corresponding code can be found at [24].

**Figure 3:**
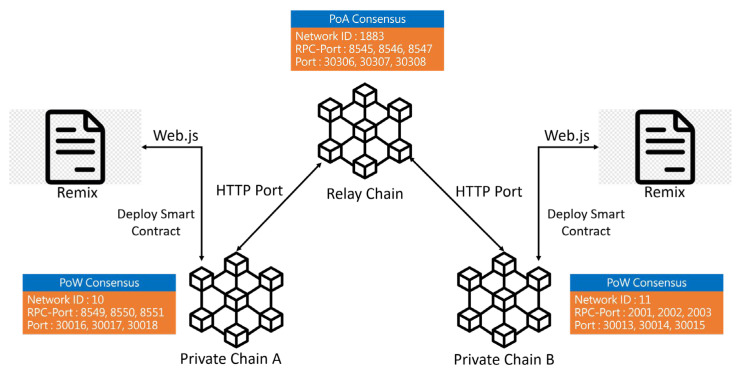
The network structure of the implementation of prototype cross-blockchain.

The network structure of our scheme is depicted in Figure 3. To simulate cross-blockchain interoperation, we constructed three blockchains, with each blockchain consisting of three nodes. Internal consensus protocols were implemented for proof-of-work (PoW) in the sub-chains and proof-of-authority (PoA) in the relay chain. The prototype cross-blockchain system was realized using Geth and Puppeth. The hardware resources utilized in our implementation included AMD Ryzen 5 5600X CPU, 32 GB DDR4 RAM, and 1 TB SATA Hard Drive. The software platforms employed were Windows 10, Remix, Solidity, Web3j CLI, Eclipse 3.8, Geth, and Puppeth. In summary, our prototype cross-blockchain system was implemented on a robust hardware and software platform, with the use of widely adopted tools and technologies in the blockchain field, to enable a realistic simulation of cross-blockchain interoperation.

In our experimental simulation, we utilized the aforementioned setup, establishing blockchains with different consensus protocols using Geth, and constructing three to nine nodes within the cross-chain system, evenly distributed across the individual blockchain systems. The system was pre-defined to tolerate two instances of erroneous behavior, which were randomly selected from Table 1. We designed a simple smart contract for the transactions, using Solidity as the programming language. Upon compilation in Remix, we transformed the application binary interface (ABI) and source code (bytecode) files into Java code. This was then integrated with our decentralized application (DAPP), which was written in Java. With each transaction initiation, the bridge node utilizes the designed smart contract to initiate the transaction. The reputation scoring is assessed at each stage via the DAPP. Within the system, our intention is to assign every node with a default reputation score of more than 10, and we aspire for the nodes to maintain a reputation score of above 8; otherwise, they will lose the right to initiate transactions.

## 5. Result and Analysis

This section mainly tests the time costs about two cross-blockchain interoperations which contain indicator reweight process and cross-chain interoperation process. The results of a number of simulations performed to measure the proposed scheme. For each interoperation, we conduct the evaluation 10 times to determine the average time cost.

### 5.1. Cross-Chain Interoperation Process

**Figure 4:**
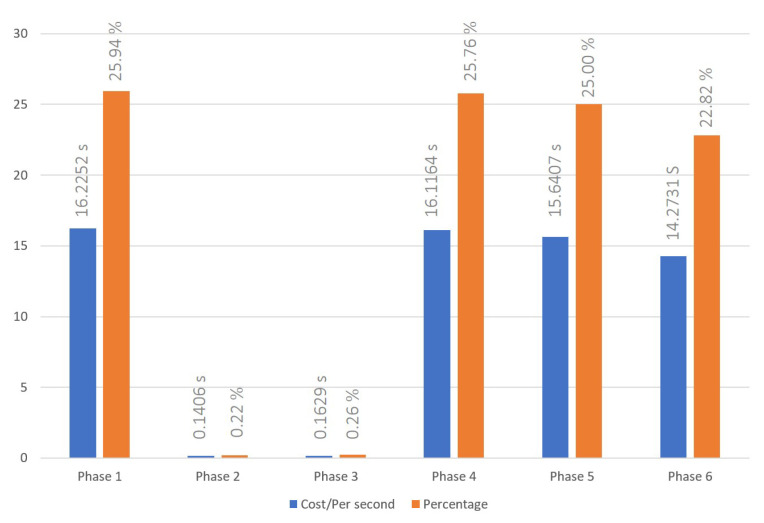
Time cost of cross-blockchain interoperation process with 3 nodes system.

**Figure 5:**
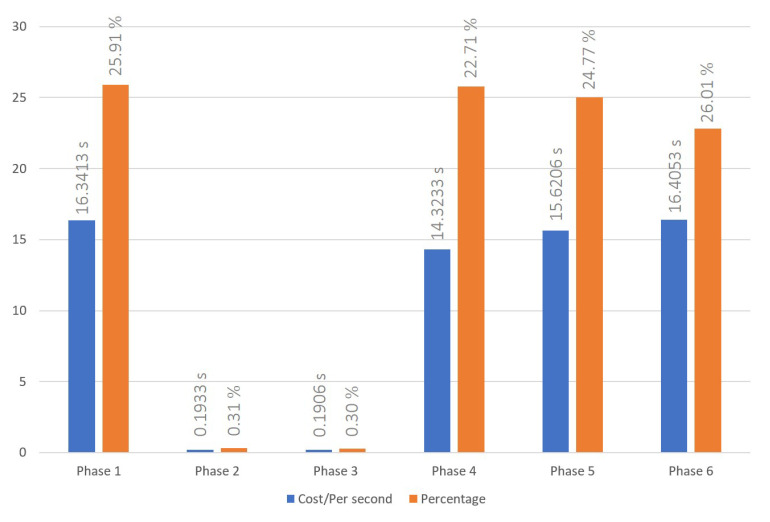
Time cost of cross-blockchain interoperation process with 6 nodes system.

**Figure 6:**
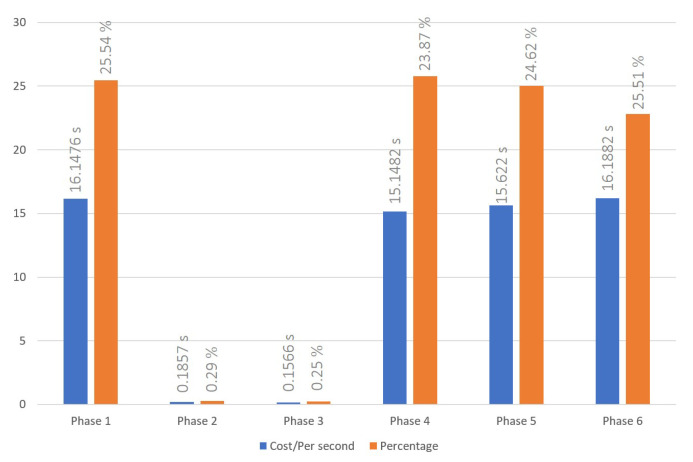
Time cost of cross-blockchain interoperation process with 9 nodes system.

**Figure 7:**
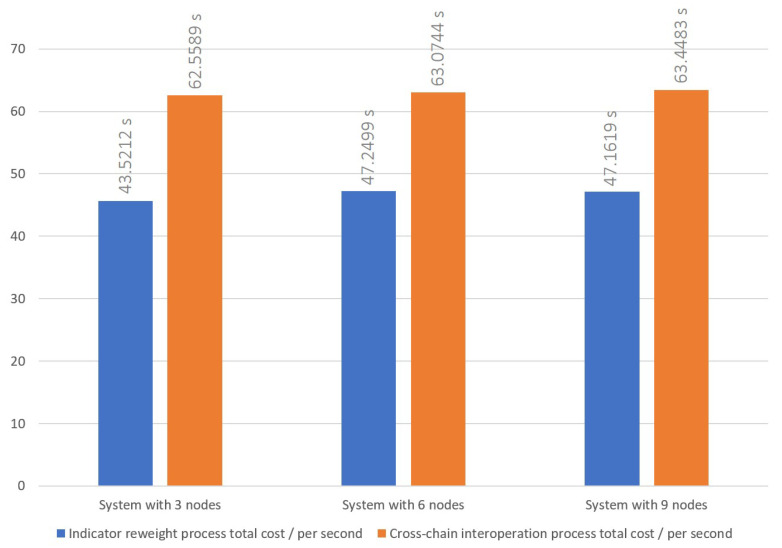
Time cost of cross-blockchain interoperation process and indicator reweight process with 3 to 9 nodes system.

The cross-chain interoperation process was conducted following the procedures outlined in Section 3.1. The time cost to successfully complete a transaction has been recorded for three, six, and nine node settings, and are shown in Figure 4, Figure 5 and Figure 6, respectively. The average times of the cross-chain interoperability process for different numbers of nodes were presented in Figure 7. The experimental results show that cross-chain interoperation process in our scheme requires 63.4484 s, on average of 10 times. In the process of cross-chain interoperation, transaction nodes make reputation evaluate whenever a transaction passes through themselves, and this reputation is based on the past transaction results and behaviors of nodes. Through phases 2 and 3, we found that when nodes assess each indicator reputation value of other nodes and chains, the average time consumed is much less than other phases. Notably, the evaluation is carried out only by the information receiving node towards the information transmitting node. Consequently, the quantity of nodes does not significantly affect the system, maintaining its efficiency irrespective of the node count.

We make cross-chain interoperation through DAPP and smart contracts, which required waiting for consensus within the blockchain at each stage before proceeding to the next stage. This approach is similar to most real-world blockchain systems, and it enables us to achieve our goal of developing a system that can be practically used in most scenarios. At the same time, we observed that most of the time costs occurred in deploying smart contracts and achieving consensus in the blockchain. As a result, we can prove that our reputation management system does not have a significant impact on the execution efficiency of cross-chain systems. The majority of time costs are determined by the original cross-chain system’s execution efficiency.

### 5.2. Indicator Reweight Process

**Figure 8:**
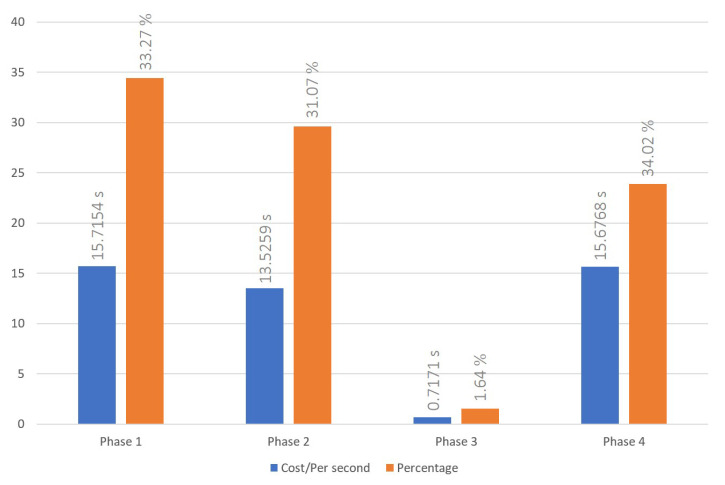
Time cost of indicator reweight process with 3 nodes system.

**Figure 9:**
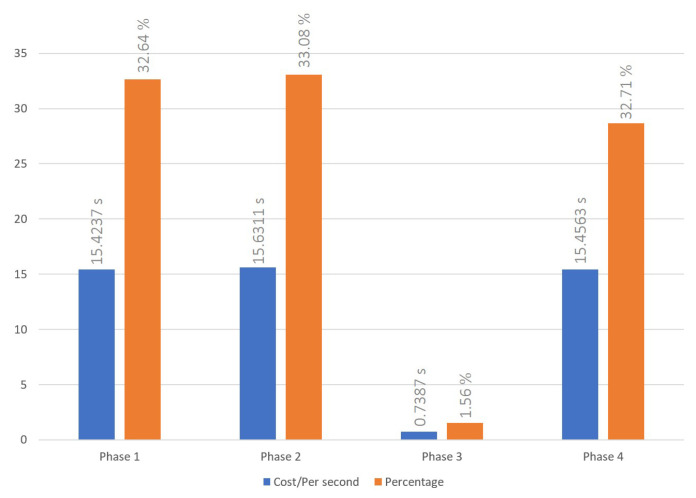
Time cost of indicator reweight process with 6 nodes system.

**Figure 10:**
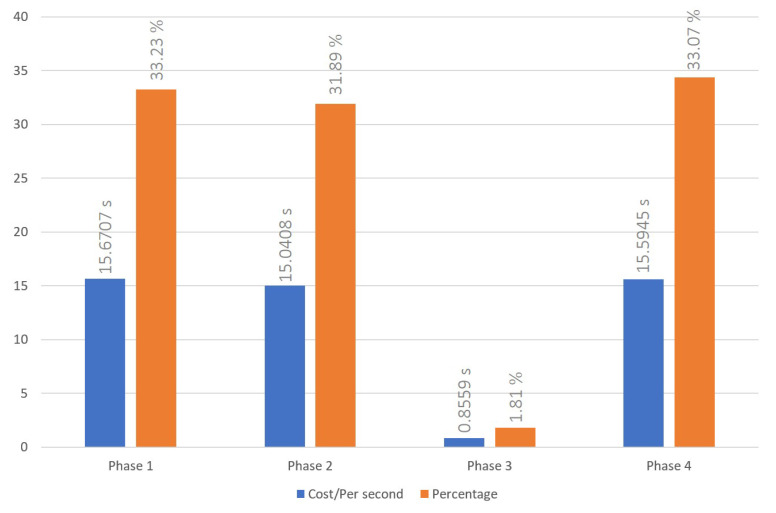
Time cost of indicator reweight process with 9 nodes system.

Our indicator reweight process was executed as depicted in Figure 8, Figure 9 and Figure 10, each figure demonstrating variations in the number of nodes. We recorded the process of a malicious node, holding fifteen reputation points, performing three malicious acts, which triggered the indicator reweighting process, as well as the time required for the adoption of the new indicator weight. Once the indicator was reweighted, the DAPP would assist in the evaluation, considering past incidents of malicious behavior and the number of tolerated occurrences. If the malicious node was successfully isolated within the number of tolerable occurrences, the reweighted indicator would be adopted. From our experimental data, we observed that, on average, the indicator reweight process under our proposed scheme takes approximately 45.6352 s over a series of 10 trials. We found that the calculation of new indicator weights during the indicator reweigh process in phase 3 is much lower than the time cost on data transmission in the other phase. This can be attributed to the following reasons. Firstly, as our reputation management system is designed to be adaptable to the majority of cross-chain scenarios, we have reduced the number of iterations in the PSO calculation process to only 10. This reduction in iterations seeks a good answer rather than the best answer. However, despite this, the newly computed indicator weights during the experimentation phase can effectively eliminate malicious nodes within the system-set rounds. Secondly, we have adopted the PoW consensus mechanism as a simulation of a private chain within the cross-chain system. During the indicator reweight process, cross-chain information transmission is facilitated through smart contracts. As discussed in the cross-chain interaction process, executing smart contracts and achieving consensus in the blockchain incurs a significant time cost. Moreover, according to Figure 7, Figure 8, Figure 9 and Figure 10, an increase in the number of nodes subtly impacts the indicator reweight process. This is primarily because, during the third stage of the indicator reweight process, the relay bridge node must await the return of node lists from all nodes in the system before proceeding.

### 5.3. Security Analysis

In our system, we perfectly integrate various indicators, which can effectively detect misbehaviors and potential attacks on blockchain [2,12,18,19,20,21,22]. Moreover, the system performance is dynamically adjusted and better improved through the PSO algorithm with different scenarios. In the following, we explain why our system can resist against the corresponding seven misbehaviors (or potential attacks) as mentioned in Section 2.3.

First, the blockchain system may encounter chain-fork (and produce stale and orphaned blocks) due to a significant gap among computation ability of entities. In that case, the system will require more propagation time to reconstruct the chain and achieve the next correct consensus. Thus, it is obvious that the average network hash rate among nodes and the delay time in block propagation can be effectively used to detect the chain-fork scenario [18]. If it happens, the system (or even administrator) may adopt appropriate countermeasures to conquer this problem. Second, dishonest miners may temporarily increase the usage ratio of their GPU computation ability to gain mining priority. In particular, these miners may keep their blocks secretly and publish it anytime. This will increase the time delay of block propagation and consensus agreement among network nodes. This scenario is called selfish mining. Hence, we utilize the total hash rate of target blockchain network, the average network hash rate among nodes and the delay time in block propagation as the effective indicators against selfish mining [12]. These three indicators are useful against time delay of block propagation and consensus agreement among nodes. Third, the above selfish miner may launch another so-called block withholding attack in which miner may provide partial information about blocks to the mining pool instead of full information to gain more rewards. To prevent this scenario, we use the hardware utilization (GPU) of nodes to detect the misusage of GPU utilization at node level, and the delay time in block propagation to detect block withholding attack at network level, respectively [19].

Fourth, all of the above misbehaviors (and attacks) may result in consensus delay in which the block validation and consensus agreement may require more time than usual. This will make the system vulnerable during transactions. Therefore, we exploit three indicators, i.e., average spending time of transaction, the delay time in block propagation and average spending time of each transaction to detect the abnormal situation of consumed time of this mis-scenario [2]. Fifth, a time-jacking attack may be launched through a series of counterfeited blocks with fake timestamp to reject new valid blocks from being included within the blockchain system. Attackers may manipulate the timestamp to perform the above attack. Hence, we choose two indicators, i.e., transaction consequence and the delay time in block propagation, to effectively detect the valid process and transmission time. This can prevent the above attacks [20]. Sixth, malicious attackers may re-exploit the used transaction when chain-fork temporarily emerges or intentionally created. This is called double-spending attack. It is obvious that the three indicators, i.e., average spending time of transaction, transaction consequence and average spending time of each transaction, are effective to detect the unstable transaction time, mis-order transaction sequence and unstable spending time for transactions [21]. This makes our system secure against double-spending attack. Seventh, a general DDoS (distributed denial of service) attack may be performed to the target blockchain network to exhaust the resource and interrupt the availability. In a DDoS scenario, the transaction consequence will be extremely unstable and overwhelming transactions may appear. Thus, it is effective to detect the DDoS attack through the transaction consequence indicator [22].

## 6. Conclusions

In this paper, we proposed a dynamic reputation management scheme used in cross-blockchain. In our scheme, we summarized several attack techniques that occur during a blockchain transaction and consider the potential factors that cause them to occur as reputation indicators. After that, we also combine the Particle Swarm Optimization (PSO) algorithm to optimize the weight of reputation indicators to better fit the different scenarios that cross-blockchain system use.

To support our research, we implement prototype cross-blockchain system with Ethereum, and each private blockchain was set as three nodes. Our proposed scheme achieved a total cost of 63.4484 s in cross-chain interoperation with nine nodes for ten times average and total cost of 47.1619 s in indicator reweight process with nine nodes for ten times average. However, the experimental results clearly show that significant time costs are consumed in phase that deploy contract or implement consensus process. Conversely, reputation judgement and the execution of the Particle Swarm Optimization (PSO) algorithm to calculate new indicator weights do not demand an excess of resources. Furthermore, these processes are not substantially influenced by the number of nodes. An increase in the number of nodes only marginally affects the efficiency of information integration by the bridge node. Hence, we consider that the time cost of cross-chain interoperation process and indicator reweight process seems to be closely connected to the performance of the adopted blockchain. The simulated cross-chain system implements the proposed dynamic reputation management scheme, integrated within the cross-chain framework, to guard against the misbehaviors and attack methodologies presently appearing in the blockchain system. Therefore, the scoring indicator for nodes have become our primary reference for effectively countering these misbehaviors.

In summary, with the aid of this dynamic reputation management scheme, we demonstrate scalability and security during cross-chain interoperation. The cross-blockchain interoperation can resist different attack techniques and frequency during real-time interoperation with dynamic indicator weights, and it also is suitable for different scenarios that the cross-blockchain system uses.

## Figures and Tables

**Table 1:** Reputation indicators and corresponding threats with condition judgement used.

Reputation Indicator	Initial Value	Condition Judgement	Pre-Define Weight	Corresponding Attack and Threat
(Node) Node connect status	None	$\left\{\begin{array}{c} if\;Async,\;-1\\ if\;sync,\;0 \end{array}\right.$	1	None
(Node) Hardware usage (GPU)	Average GPU computing power of a period of time.	$\left\{\begin{array}{c} Increase\;or\;Decrease\;rapidly,\;-1\\ Slow\;or\;Constant,\;0 \end{array}\right.$	0.6	Selfish mining, Block withholding, Majority attack
(Node) Average spending time of transaction	Expect time $E\left(T\right)=\frac{Difficulty\_value}{{Hash\_rate}_{}}$	$\left\{\begin{array}{c} Overtime,\;-1\\ Ontime,\;0\\ Less\;time\;consumption,\;-1 \end{array}\right.$	0.4	Double-spending, Consensus delay
(Node) Transaction consequence	None	$\left\{\begin{array}{c} Success,\;1\\ Failure,\;-1 \end{array}\right.$	1	DDoS, Double-spending, Time-jacking attack
(Chain) Average network hash rate	Average network hashing power of a period time	$\left\{\begin{array}{c} Increase\;or\;Decrease\;rapidly,-1\\ Slow\;or\;Constant,\;0 \end{array}\right.$	0.4	Stale and orphaned blocks, Selfish mining, Majority attack
(Chain) The delay time in block propagation	None	$\left\{\begin{array}{c} Delay\;\frac{E\left(T\right)}{2}\;time,\;-1\\ On\;time,\;0 \end{array}\right.$	1	Consensus delay, Selfish mining, Block withholding, Stale and orphaned blocks, Time-jacking attacks
(Chain) Average spending time of each transaction	Expect time $E\left(T\right)=\frac{Difficulty\_value}{{Hash\_rate}_{}}$	$\left\{\begin{array}{c} Overtime,\;-1\\ On\;time,\;0\\ Less\;time\;consumption,\;-1 \end{array}\right.$	0.4	Double-spending, Consensus delay

**Table 2:** Implement environment.

Item	Description
Hardware Resources	AMD Ryzen 5 5600X 6-Core Processor 3.70 GHz ASUS DUAL-RTX3060TI-O8G-V2 Crucial Ballistix 32 GB 3200 MHz DDR4 RAM WD2003FZEX-00SRLA0 1 TB SSD
Software Platform	Windows 10 64 Bit
	Ethereum GO 1.16.3 Solidity 0.4.21 Remix IDE Web3.js Oracle Java 17 Eclipse 3.8 with Open JDK Geth 1.9.25 with Puppeth

## Data Availability

Not applicable.
